# A fact‐finding survey on self‐efficacy of foot care behaviour in patients with diabetes: Analysis using the Diabetes Study from the Center of Tokyo Women’s Medical University 2017 (DIACET 2017)

**DOI:** 10.1002/edm2.219

**Published:** 2020-12-19

**Authors:** Kazuki Ikura, Hayato Kato, Haruna Azuma, Yuri Oda, Yuka Kato, Junnosuke Miura, Tetsuya Babazono

**Affiliations:** ^1^ Diabetes Center Tokyo Women’s Medical University School of Medicine Tokyo Japan

**Keywords:** diabetes, foot care, foot care confidence scale, self‐care, self‐efficacy

## Abstract

**Aim:**

We aimed to determine the association between self‐efficacy of foot care behaviour and chronic complications in Japanese patients with diabetes.

**Methods:**

We conducted a cross‐sectional study based on a questionnaire survey of 4571 patients with type 1 and type 2 diabetes who had (a) given consent to participate in the Diabetes Study from the Center of Tokyo Women's Medical University: DIACET 2017, and (b) completed all the questions of the Japanese Version of Foot Care Confidence Scale (J‐FCCS), consisting of 12 statements.

**Results:**

A greater proportion of respondents answered that they were not confident in determining the condition of corns and/or calluses and the condition of toenails. The J‐FCCS total scores of the patients with retinopathy (*p* <.001) and numbness or pain in the feet (*p* <.001) were significantly lower than those of the patients without these complications. In both the multiple regression analysis and logistic regression analysis, lower J‐FCCS was significantly associated with retinopathy and numbness or pain in the feet.

**Conclusion:**

Foot care education that emphasizes a psychological approach in improving confidence associated with foot self‐care is important for patients with advanced complications of diabetes.

## Graphical abstract

We conducted this large study to clarify the association between self‐efficacy of foot care behaviour and microvascular complications in patients with type 1 and type 2 diabetes, using the Japanese version of the FCCS (J‐FCCS). The multiple regression analysis showed that retinopathy and numbness or pain in the feet, but not CKD, were significantly associated with lower J‐FCCS total scores.

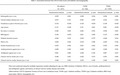

## 1 INTRODUCTION

Diabetic foot ulcers are a major cause of non‐traumatic lower extremity amputations in patients with diabetes,^1^ which is associated with increased mortality.^2^, ^3^ Therefore, prevention of foot ulcers is a critical issue for patients with diabetes to improve their survival. Prophylactic foot care to prevent foot ulcers includes identification and periodical assessment of high‐risk patients, treatment of pre‐ulcerative lesions, instruction to use of appropriate footwear and education of foot care, especially foot self‐care.^4^ In foot care education, it is important for healthcare providers to motivate patients to care of themselves and guide them to re‐evaluate their daily behaviours by themselves so that they can start introducing changes in their daily lifestyles. To initiate this behaviour modification, patients need not only acquisition of knowledge and skills related to foot care, but also improvement of patient self‐efficacy as a psychological approach. Self‐efficacy is belief that what one intends to do is efficacious and confidence to properly conduct the behaviour.^5^

Educational interventions targeting self‐efficacy improvement in patients with diabetes have been recently reported to promote the aggressiveness of foot self‐care behaviours.^6^, ^7^, ^8^ The Foot Care Confidence Scale (FCCS)^9^ which evaluates self‐efficacy of foot care behaviour has been recently utilized as an effect measurement after foot care education programs.^10^, ^11^ While foot care education with an awareness of self‐efficacy is required in Japan, there are very few studies on self‐efficacy of foot care behaviour in patients with diabetes, the association between self‐efficacy of foot care behaviour and clinical background has not been thoroughly investigated.^12^, ^13^ In addition, the sample size of these studies were small, limiting the generalizability. We therefore conducted this large study to clarify the association between self‐efficacy of foot care behaviour and microvascular complications in patients with type 1 and type 2 diabetes, using the Japanese version of the FCCS (J‐FCCS).^13^

## 2 MATERIAL AND METHODS

### 2.1 Study design and ethical issues

This was a single‐centre cross‐sectional study that was approved by the Ethics Committee of Tokyo Women's Medical University (Approval No. 2481‐R2), in compliance with the Declaration of Helsinki, paying utmost attention specifically to the protection of the participant privacy.

### 2.2 Subjects

The subjects were Japanese patients with type 1 and type 2 diabetes who had (a) given consent to participate in the Diabetes Study from the Center of Tokyo Women's Medical University: DIACET 2017, an observational study on the current status of diabetes treatment, starting in October 2017, and (b) completed all the questions of the J‐FCCS. Subjects with normal glucose tolerance, borderline diabetes and other types of diabetes were excluded. Patients who had withdrawn from insulin therapy after successful pancreas transplantation were included in patients with type 1 diabetes.

### 2.3 Methods

As described previously,^14^ self‐administered questionnaires were distributed to all outpatients visiting our centre and in patients at admission to investigate the subject's status of glycemic control, subjective symptoms related to diabetic complications and history of clinical visit for cardiovascular diseases, as well as self‐efficacy of foot care behaviour using the J‐FCCS. Laboratory data and information on the presence or absence of any stage of diabetic retinopathy and nephropathy were collected from medical records.

The FCCS questionnaire includes 12 statements related to undertaking various foot care behaviour (Table 1), and the subjects were asked to rate the degree of self‐efficacy. A five‐point scale was scored from 1 to 5, each of which corresponds to ‘strongly not confident’, ‘moderately not confident’, ‘confident’, ‘moderately confident’ and ‘strongly confident. The total score consisting of the 12 statements ranging 12 to 60 was then calculated. Higher scores indicates higher levels of self‐efficacy of foot care behaviour. The validity and reliability of the Japanese version, J‐FCCS, have been demonstrated in the past.^13^

**TABLE 1:** The J‐FCCS statements

		Strongly not confident	Moderately not confident	Confident	Moderately confident	Strongly confident
Q1	I can protect my feet	2.8%	7.5%	25.4%	35.5%	28.8%
Q2	Even without pain/discomfort, I can look at my feet daily to check for cuts, scratches, blisters, redness, or dryness	2.6%	5.7%	15.1%	39.7%	36.9%
Q3	After washing my feet, I can dry between my toes	2.3%	5.1%	12.4%	35.0%	45.2%
Q4	I can judge when my toenails need to be trimmed by a podiatrist	5.0%	7.3%	17.4%	29.6%	40.7%
Q5	I can trim my toenails straight across	4.5%	5.7%	8.7%	32.9%	48.2%
Q6	I can figure out when to use a pumice stone to smooth corns and/or calluses on my feet	8.6%	9.7%	23.7%	26.1%	31.9%
Q7	I can test the temperature of the water before putting my feet into it	1.7%	1.9%	7.9%	33.4%	55.1%
Q8	If I was told to do so, I can wear shoes and socks every time I walk (includes walking indoors)	1.2%	1.9%	5.8%	29.0%	62.0%
Q9	When I go shopping for new shoes, I can choose shoes that are good for my feet	0.9%	2.4%	7.0%	31.5%	58.2%
Q10	I can call my doctor about problems with my feet	1.0%	2.9%	12.5%	33.9%	49.7%
Q11	Before putting them on, I can check the insides of my shoes for problems that could harm my feet	1.3%	2.7%	9.5%	31.4%	55.0%
Q12	If directed to do so, I can routinely apply lotion to my feet	0.8%	2.9%	7.8%	33.6%	54.8%

Abbreviations: J‐FCCS, Japanese Version of Foot Care Confidence Scale

For haemoglobin A1c (HbA1c) and serum creatinine, mean levels measured between January and December 2017 were used. The estimated glomerular filtration rate (eGFR) was calculated based on serum creatinine levels, age and sex.^15^ An eGFR <60 ml/min/1.73 m^2^ or being on dialysis therapy was defined as chronic kidney disease (CKD).^16^

### 2.4 Statistical analysis

Continuous variables were expressed as arithmetic mean ± standard deviation (SD) or geometric mean with 95% CI, as appropriate according to data distribution. Categorical data were expressed by number (%). Continuous data were compared using Student's *t* test and categorical data using Fisher's exact test. Analysis of covariance (ANCOVA) were used to compare the total J‐FCCS scores by type of diabetes. Based on the median of the total J‐FCCS score, the patients were classified into higher and lower J‐FCCS score groups. The associations between J‐FCCS scores and diabetic complications were examined using the multiple regression analysis and logistic regression analysis adjusting for the following parameters: age, sex, body mass index (BMI), HbA1c, duration of diabetes, use of insulin, antihypertensive and antilipemic agents, and history of clinical visit for cardiovascular diseases. *p* values <.05 were considered significant. All statistical analyses were performed using the SAS version 9.4 (SAS Institute, Cary, NC, USA).

## 3 RESULTS

### 3.1 Clinical characteristics of the study subjects

**FIGURE 1:**
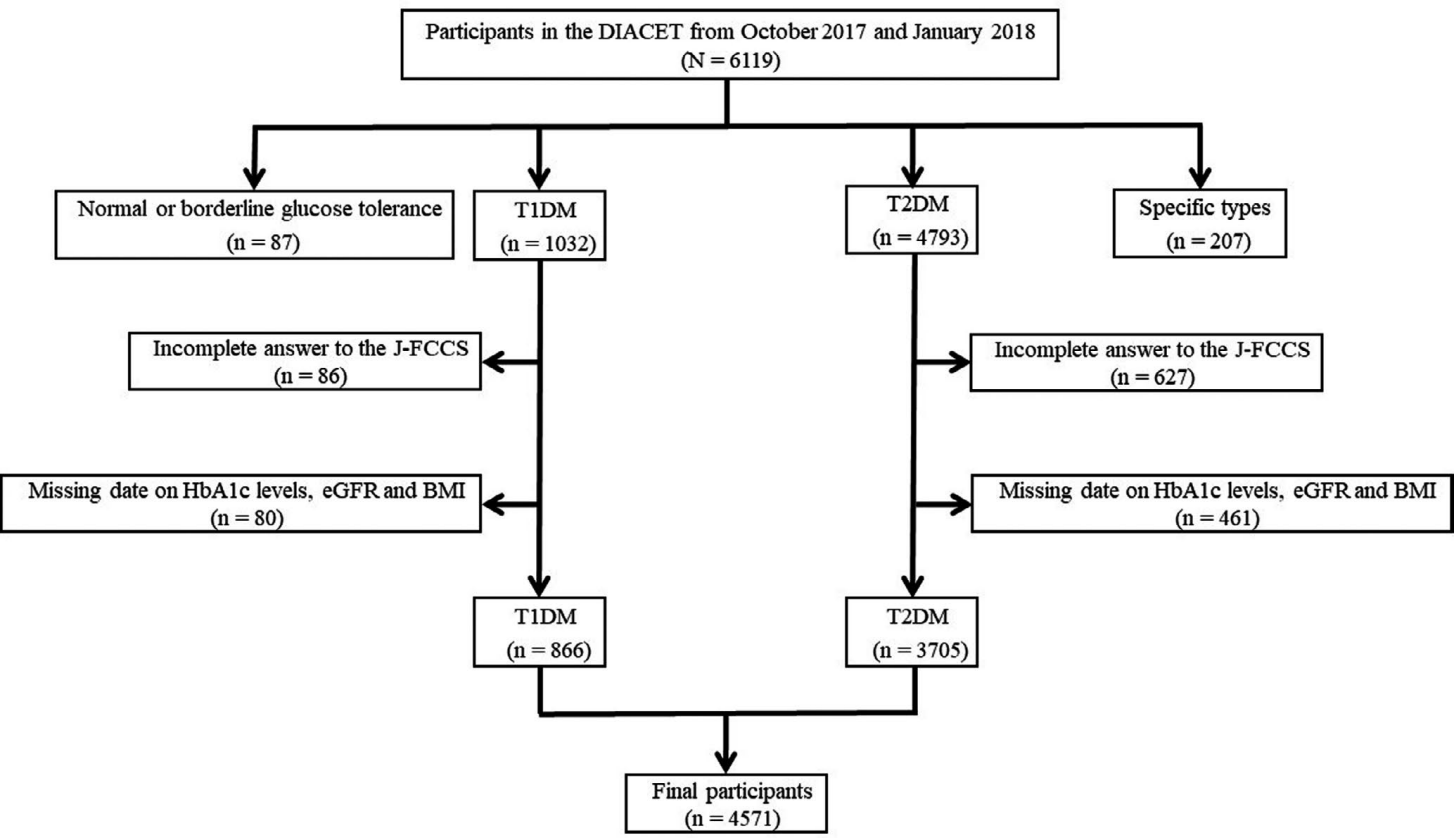
Flow chart showing the number of study participants. DIACET, the Diabetes Study from the Center of Tokyo Women's Medical University; T1DM, type 1 diabetes mellitus; T2DM, type 2 diabetes mellitus; J‐FCCS, Japanese Version of Foot Care Confidence Scale; HbA1c, haemoglobin A1c; eGFR, estimated glomerular filtration rate; BMI, body mass index

From the overall 7333 participants registered in the DIACET, we obtained 6119 responses with the response rate of 83.4%. Among 6119 patients, individuals with normal or borderline glucose tolerance as well as patients with diabetes other than type 1 and type 2 were excluded. Next, from the remaining 5825 patients with type 1 and type 2 diabetes, those who did not answer more than one of the 12 questions in the J‐FCCS (*N* = 713) and those with missing date on HbA1c levels, eGFR and BMI (*N* = 541) were eliminated. Finally, a total of 4571 patients (mean [±SD] age 63 ± 15 years; 2630 men, 1941 women; 866 patients with type 1 diabetes and 3705 patients with type 2 diabetes) were included in this study (Figure 1). Table 2 shows the clinical characteristics of overall subjects and those classified by type of diabetes. Patients with type 1 diabetes were younger (*p* < .001), included more women (*p* < .001) and had a longer diabetes duration (*p* < .001) than those with type 2 diabetes. Patients with type 2 diabetes were more likely to have hypertension, dyslipidemia, and micro‐ and macroangiopathic complications.

**TABLE 2:** Clinical characteristics and laboratory data

	All subjects (*N* = 4571)	T1DM (*N* = 866)	T2DM (*N* = 3705)	*p* value
Age (years)	63.0 ± 14.5	48.3 ± 15.1	66.4 ± 12.0	<.001
Men (%)	57.5	31.1	63.7	<.001
BMI (kg/m^2^)	24.4 ± 4.2	23.3 ± 3.6	24.7 ± 4.3	<.001
Diabetes duration (years)	20.0 ± 11.5	22.3 ± 12.5	19.5 ± 11.2	<.001
HbA1c (%)	7.5 ± 1.1	7.8 ± 1.1	7.5 ± 1.1	<.001
Insulin treatment (%)	49.3	98.5	37.8	<.001
Antihypertensive agents (%)	49.4	31.4	53.6	<.001
Antilipemic agents (%)	43.5	24.3	48.0	<.001
Retinopathy (%)	40.5	35.9	41.6	.002
Dialysis (%)	0.9	0.8	1.0	.845
Numbness and pain in the feet (%)	59.8	47.3	62.8	<.001
Clinical visit for stroke (%)	3.2	1.2	3.7	<.001
Clinical visit for cardiac disease (%)	19.1	7.6	21.8	<.001
J‐FCCS total score	50.0 ± 8.6	49.5 ± 8.3	50.2 ± 8.6	.045

Note

Data are expressed as number (%) or mean ± SD. Clinical findings between the two types of diabetes were compared using Student's *t* test for continuous data and Fisher's exact test for categorical data.

Abbreviations: T1DM, type 1 diabetes mellitus; T2DM, type 2 diabetes mellitus; BMI, body mass index; HbA1c, haemoglobin A1C; J‐FCCS, Japanese Version of Foot Care Confidence Scale; SD, standard deviation.

### 3.2 Percentages for each of the J‐FCCS statements

Table 1 shows the percentages of patients responding to each J‐FCCS statement evaluated on a five‐point scale. Of the 12 statements, that with the greatest proportion of the subjects responding ‘strongly or moderately not confident’ was ability to identify the condition of corns and calluses (Question 6), followed by ability to identify the condition of the toenails (Question 4).

### 3.3 Comparison of the J‐FCCS total scores by type of diabetes and sex

The median (range) and mean (±SD) J‐FCCS total score of overall subjects was 51 (12–60) and 50.0 ± 8.6, respectively. The difference of the mean J‐FCCS total scores for patients with type 1 and type 2 patients was minimal but statistically significant (Table 1). After adjustment for the above confounders, the significance of the J‐FCCS score disappeared (*p* = .300). There was no significant difference in the J‐FCCS total scores between men and women (*p* = .091).

### 3.4 The association between the J‐FCCS total scores and diabetic microangiopathy

**FIGURE 2:**
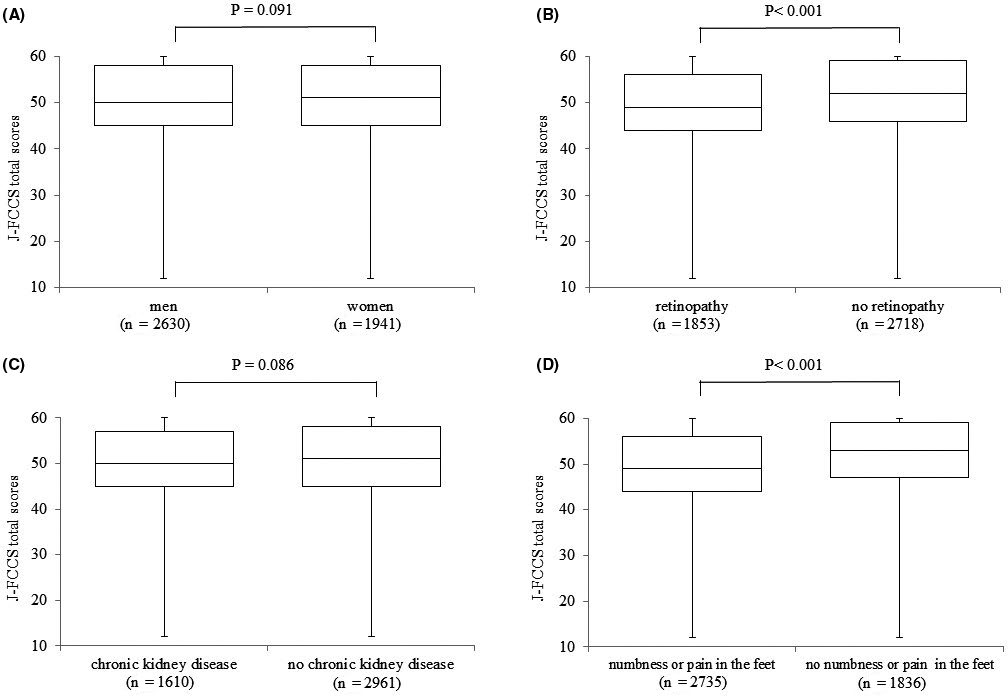
Comparison of the J‐FCCS total scores by sex (A), presence or absence of diabetic retinopathy (B), chronic kidney disease (C) and numbness or pain in the feet associated with neuropathy (D) using Student's *t* test. J‐FCCS, Japanese Version of Foot Care Confidence Scale; eGFR, estimated glomerular filtration rate

The scores of the patients with microvascular complications were significantly lower than those of the patients without (Figure 2, retinopathy [*p* < .001], CKD [*p* =.086], and numbness or pain in the feet associated with neuropathy [*p* < .001]). The multiple regression analysis showed that retinopathy (*p* < .001) and numbness or pain in the feet (*p* < .001), but not CKD (*p* = .118), were significantly associated with lower J‐FCCS total scores (Table 3). In the logistic regression analysis, presence of retinopathy and numbness or pain in the feet were also significantly associated with lower J‐FCCS score (Table 4). The same trends were observed in the separate analysis by type of diabetes (Tables 3 and 4).

**TABLE 3:** Association between the J‐FCCS total scores and diabetic microangiopathy.

	All subjects (*N* = 4571)		T1DM (*N* = 866)		T2DM (*N* = 3705)	
	Standardized estimate	*p* value	Standardized estimate	*p* value	Standardized estimate	*p* value
Retinopathy (yes vs no)	−0.059	.001	−0.085	.034	−0.054	.002
Chronic kidney disease (yes vs no)	−0.015	.331	0.020	.593	−0.016	.364
Numbness or pain in the feet (yes vs no)	−0.154	<.001	−0.165	<.001	−0.149	<.001
Age (years)	0.119	<.001	0.151	.001	0.081	<.001
Sex (men vs women)	−0.046	.002	0.004	.916	−0.056	.001
BMI (kg/m^2^)	−0.100	<.001	0.001	.990	−0.127	<.001
Diabetes duration (years)	−0.040	.019	−0.023	.579	−0.046	.016
HbA1c (%)	−0.009	.564	−0.019	.598	−0.005	.766
Insulin treatment (yes vs no)	−0.052	.002	0.025	.462	−0.053	.003
Antihypertensive agents (yes vs no)	−0.008	.592	−0.002	.963	−0.009	.591
Antilipemic agents (yes vs no)	0.002	.882	−0.049	.169	0.007	.657
Clinical visit for stroke (yes vs no)	−0.048	.001	−0.022	.515	−0.051	.002
Clinical visit for cardiac disease (yes vs no)	−0.048	.001	−0.033	.349	−0.045	.005

Note

Data were analysed using the multiple regression models adjusting for age, sex, BMI, duration of diabetes, HbA1c, use of insulin, antihypertensive and antilipemic agents, and history of clinical visit for cardiac disease and stroke.

Abbreviations: J‐FCCS, Japanese Version of Foot Care Confidence Scale; T1DM, type 1 diabetes mellitus; T2DM, type 2 diabetes mellitus; BMI, body mass index; HbA1c, haemoglobin A1c.

**TABLE 4:** Association between low J‐FCCS scores (50 or less) and diabetic microangiopathy

	All subjects (*N* = 4571)				T1DM (*N* = 866)				T2DM (*N* = 3705)			
	Univariate		Multivariate		Univariate		Multivariate		Univariate		Multivariate	
	OR (95%CI)	*p* value	OR (95% CI)	*p* value	OR (95% CI)	*p* value	OR (95% CI)	*p* value	OR (95% CI)	*p* value	OR (95% CI)	*p* value
Retinopathy (yes vs no)	1.49 (1.33–1.68)	<.001	1.20 (1.05–1.38)	.007	1.56 (1.18–2.07)	.002	1.37 (0.98–1.93)	.069	1.49 (1.31–1.70)	<.001	1.19 (1.02–1.38)	.024
Chronic kidney disease (yes vs no)	1.13 (1.00–1.27)	.057	1.07 (0.93–1.22)	.372	1.07 (0.76–1.51)	.713	0.94 (0.63–1.40)	.740	1.18 (1.03–1.34)	.017	1.06 (0.91–1.23)	.449
Numbness or pain in the feet (yes vs no)	1.70 (1.51–1.91)	<.001	1.68 (1.48–1.91)	<.001	1.84 (1.41–2.42)	<.001	1.87 (1.40–2.50)	<.001	1.72 (1.51–1.97)	<.001	1.64 (1.42–1.89)	<.001
Age (per 1 years)			0.99 (0.98–0.99)	<.001			0.99 (0.98–0.99)	.013			0.99 (0.98–0.99)	.014
Sex (men vs women)			1.23 (1.09–1.40)	.001			1.21 (0.89–1.63)	.222			1.26 (1.09–1.45)	.001
BMI (per 1.0 kg/m^2^)			1.04 (1.02–1.05)	<.001			0.99 (0.95–1.03)	.649			1.05 (1.03–1.07)	<.001
Diabetes duration (per 1 year)			1.01 (1.01–1.02)	.001			1.01 (0.99–1.02)	.341			1.01 (1.01–1.02)	.001
HbA1c (per 1.0%)			1.04 (0.98–1.11)	.157			1.06 (0.93–1.20)	.410			1.04 (0.97–1.11)	.318
Insulin treatment (yes vs no)			1.20 (1.05–1.38)	.008			0.74 (0.22–2.44)	.619			1.18 (1.01–1.37)	.035
Antihypertensive agents (yes vs no)			0.99 (0.87–1.13)	.867			1.05 (0.75–1.49)	.767			0.97 (0.84–1.12)	.700
Antilipemic agents (yes vs no)			0.98 (0.86–1.11)	.712			1.09 (0.77–1.53)	.630			0.98 (0.86–1.12)	.769
Clinical visit for stroke (yes vs no)			1.66 (1.17–2.36)	.004			1.13 (0.31–4.17)	.855			1.69 (1.18–2.43)	.005
Clinical visit for cardiac disease (yes vs no)			1.23 (1.05–1.44)	.009			1.13 (0.66–1.93)	.658			1.22 (1.04–1.44)	.016

Note

Data were analysed using the logistic regression models adjusting for age, sex, BMI, duration of diabetes, HbA1c, use of insulin, antihypertensive and antilipemic agents, and history of clinical visit for cardiac disease and stroke.

Abbreviations: J‐FCCS, Japanese Version of Foot Care Confidence Scale; T1DM, type 1 diabetes mellitus; T2DM, type 2 diabetes mellitus; BMI, body mass index; HbA1c, haemoglobin A1C; OR, odds ratio; CI, confidence interval.

## 4 DISCUSSION

The aim of the present study was to determine the association between self‐efficacy of foot care behaviour and microvascular complications in a large sample of Japanese patients with type 1 and type 2 diabetes. Of the 12 questions of the J‐FCCS, greater proportion of the subjects who reported lack of confidence were those related to determining the condition of corns, calluses or toenails. The total J‐FCCS score in this study was higher than that in another Japanese study.^13^ We also found that diabetic retinopathy and neuropathy were significantly associated with the J‐FCCS scores in both types of diabetes.

The lack or lower confidence in identifying the condition of corns, calluses and toenails in many patients was also reported by another Japanese group.^12^ This may be due to the difficulty in handling corns and calluses by themselves in patients who have never had these foot lesions. Calluses are formed when the skin is repeatedly subjected to mechanical stimuli, such as compression and friction. When left untreated, further pressure is applied to cause ulcers. Therefore, regular removal of calluses and assessment of plantar pressure are needed to prevent recurrence. In patients with foot deformities, shoes and insoles fitted to the shape of the foot are needed.^17^, ^18^ To improve self‐efficacy and prevent the development of foot ulcers, a better understanding of corns and calluses should be provided, as well as guidance on daily careful observation of the feet.

Many patients with diabetes have nail abnormalities. Severely thickened and deformed nails, ingrown nails, and tinea unguium make it difficult to cut nails by themselves. Furthermore, in patients with impaired vision due to proliferative diabetic retinopathy, their skin may be accidentally cut with a nail cutter, causing foot ulcers. In patients who lack confidence in cutting their own nails, nail care by healthcare stuffs may be needed.

The median of the J‐FCCS total score for all 4571 study subjects was 51, which was higher than that reported by Matsumoto et al^13^, who investigated the validity and reliability of the Japanese version of the FCCS in patients with diabetes. One explanation could be related to the six‐day‐a‐week outpatient clinic allocated at our hospital for foot care. In addition, our hospital has a dedicated outpatient nursing clinic run exclusively by experienced diabetes nurses. If a patient or the attending physician finds abnormalities in the foot, podiatrists in the foot care can see the patient immediately in our hospital. Patients are then given guidance about foot care. The present study findings may have been affected by the above. A study found that performing a five‐minute risk assessment with a 15‐min self‐care guidance session for outpatients significantly increased self‐efficacy one month later and enhanced the willingness of the patients to perform foot self‐care behaviour.^6^ Guidance including hands‐on practice during outpatient visits may be effective in increasing self‐efficacy of foot care behaviour in patients with diabetes.

The association between diabetic microangiopathy and self‐efficacy of foot care behaviour has not been clarified. Since this was a cross‐sectional observational study, the causal relationship between self‐efficacy of foot care behaviour and diabetic microangiopathy was not clarified. However, studies have found that many patients with diabetes with retinopathy and neuropathy did not engage in foot self‐care behaviour.^19^, ^20^ From the present study, the reason may be associated with the lack of confidence in engaging in foot care behaviour due to visual impairment and sensory impairment of the skin. A study of patients with diabetic peripheral neuropathy found an association between daily foot observations and self‐efficacy,^21^ suggesting that foot care education that emphasizes a psychological approach to improve confidence associated with foot self‐care may be needed in patients with advanced complications of diabetes.

Even with high self‐efficacy, patients may not engage in self‐care behaviour if they themselves do not sufficiently feel the need.^22^ In addition to self‐efficacy, engagement of self‐care behaviour is reported to be associated with a high level of knowledge about diabetes, social support, and advice from family members and healthcare providers.^21^, ^23^ Therefore, it is important that healthcare providers (a) carefully and continuously explain the need for self‐care, (b) motivate the patients, (c) check that self‐care has been implemented and (d) conduct regular self‐efficacy assessments. In this respect, we believe that the use of the J‐FCCS, which quickly and conveniently checks the state of mind related to foot care behaviour, is useful.

The limitations of the study were as follows. Since this study was conducted at a single university hospital, the subjects may not be representative of Japanese patients with diabetes. Selection bias cannot be ruled out because subjects in DIACET was voluntary, and the present study also investigated self‐efficacy of foot care behaviour using a self‐administered questionnaire. Therefore, subjects who were unable to complete the questionnaire, due for example to severe visual impairment and dementia, were not included, probably overestimating the results. Furthermore, there may be potential factors that affected self‐efficacy, such as the assessment of blood flow in the lower extremities and clinical characteristics including lipids, blood pressure, smoking history, alcohol consumption and a history of diabetic foot ulcers. The presence of numbness or pain in the feet associated with neuropathy was also determined based on self‐reporting by the patient. Conditions that trigger numbness and pain in the feet include spinal disorders such as herniated intervertebral disc and peripheral arterial diseases. It is desirable to conduct a comprehensive neuropathy assessment by testing vibration and pressure sense and the Achilles tendon reflex. Lastly, since this was a cross‐sectional observational study, the causal relationship between self‐efficacy of foot care behaviour and diabetic complications remains unknown. Longitudinal studies are needed to assess causal association between self‐efficacy of foot care behaviour and diabetic complications.

## 5 CONCLUSION

The present fact‐finding survey showed that a large proportion of Japanese patients with diabetes were not confident in determining the condition of corns, calluses and toenails. Lower self‐efficacy of foot care behaviour was strongly associated with diabetic retinopathy and neuropathy. Foot care education that emphasizes a psychological approach in improving confidence associated with foot self‐care is considered to be important for patients with advanced complications of diabetes. Regular self‐efficacy assessments using the J‐FCCS are needed to improve self‐efficacy and prevent the development of foot ulcers.

## CONFLICT OF INTEREST

The authors declare that they have no competing interest.

## AUTHOR CONTRIBUTIONS

K.I. conceived of the study, designed the protocol, contributed to data collection and preparation, analysed all data, wrote the manuscript, contributed to the interpretation of the results and approved the final version. H.K., H.A., Y.O. and Y.K. contributed to data collection and preparation, contributed to the interpretation of the results and approved the final version. J.M. and T.B. designed the protocol, analysed all data, wrote the manuscript, contributed to the interpretation of the results and approved the final version. T.B. is the guarantor of this work.

## Data Availability

The data that support the findings of this study are available from the corresponding author upon reasonable request.
